# Waveguide Bragg Gratings in Ormocer^®^s for Temperature Sensing

**DOI:** 10.3390/s17112459

**Published:** 2017-10-26

**Authors:** Maiko Girschikofsky, Manuel Rosenberger, Michael Förthner, Mathias Rommel, Lothar Frey, Ralf Hellmann

**Affiliations:** 1 Applied Laser and Photonics Group, University of Applied Sciences Aschaffenburg, 63743 Aschaffenburg, Germany; manuel.rosenberger@h-ab.de (M.R.); ralf.hellmann@h-ab.de (R.H.); 2 Chair of Electron Devices, Friedrich-Alexander University Erlangen, 91058 Erlangen, Germany; michael.foerthner@leb.eei.uni-erlangen.de (M.F.); lothar.frey@leb.eei.uni-erlangen.de (L.F.); 3 Fraunhofer Institute for Integrated Systems and Device Technology (IISB) Erlangen, 91058 Erlangen, Germany; mathias.rommel@iisb.fraunhofer.de

**Keywords:** Bragg grating, temperature, humidity, hybrid polymer, Ormocer^®^

## Abstract

Embedded channel waveguide Bragg gratings are fabricated in the Ormocer^®^ hybrid polymers OrmoComp^®^, OrmoCore, and OrmoClad by employing a single writing step technique based on phase mask technology and KrF excimer laser irradiation. All waveguide Bragg gratings exhibit well-defined reflection peaks within the telecom wavelengths range with peak heights of up to 35 dB and −3 dB-bandwidths of down to 95 pm. Furthermore, the dependency of the fabricated embedded channel waveguide Bragg gratings on changes of the temperature and relative humidity are investigated. Here, we found that the Bragg grating in OrmoComp^®^ is significantly influenced by humidity variations, while the Bragg gratings in OrmoCore and OrmoClad exhibit linear and considerably high temperature sensitivities of up to −250 pm/${}^{\circ }$C and a linear dependency on the relative humidity in the range of −9 pm/%.

## 1. Introduction

Fiber Bragg gratings (FBGs) are a sinusoidal refractive index variation within a fiber core, and are well known to serve as efficient optical sensor elements [1,2,3]. Both the effective refractive index of the guided mode *n*${}_{\mathrm{eff}}$ as well as the grating period Λ depend on multiple external influences, such as distortion or temperature [4,5]. With respect to the Bragg grating condition (1), a change in any of these parameters alters the narrow-band Bragg wavelength $\lambda_{B}$ that is reflected from the Bragg grating.

(1) $$\lambda_{B}=2\cdot n_{\mathrm{eff}}\cdot \Lambda$$

Because of their light and thin design, their immunity to electromagnetic interference, and their small signal attenuation which allows long-distance interrogations [3], combined with their almost ideal linear dependency to temperature changes [6], fiber Bragg gratings represent one of the most discussed and employed concepts for optical temperature sensing [7]. Commonly, FBG temperature sensors are fabricated within silica-based single-mode optical fibers [5], and exhibit a temperature-dependent Bragg wavelength shift of approx. $\Delta \lambda_{B}$/Δθ = 13 pm/${}^{\circ }$C which is (as shown in relation (2)) predefined and limited by the thermo-optic coefficient α (TOC) and the coefficient of thermal expansion ζ (CTE) of the fiber material [8].

(2) $$\Delta \lambda_{B}=\lambda_{B}\left(\alpha +\zeta \right)\Delta \theta$$

Within relation (2), the TOC can be expressed as α = $\partial \Lambda /\partial \theta \cdot \Lambda {}^{-1}$, and the CTE as ζ = $\partial n_{\mathrm{eff}}/\partial \theta \cdot n_{\mathrm{eff}}^{-1}$. In order to enhance the temperature sensitivity of FBGs, the Bragg gratings can be coated with or directly written in a material — either fiber-based or as planar waveguide design — that features a higher TOC or CTE. By this measure, a sensitivity increase of typically three-to-ten-times the sensitivity of silica-based Bragg gratings can be achieved [9,10,11,12,13,14].

Due to their high (although negative) thermo-optic coefficient, polymers represent a promising material for the fabrication of highly sensitive Bragg grating-based temperature sensors. However, a major drawback of polymeric materials lies in their reduced chemical and thermal stability, thus limiting their field of use. Here, hybrid polymers such as the inorganic–organic Ormocer^®^s OrmoComp^®^, OrmoCore, and OrmoClad represent an adequate alternative. These duroplast materials exhibit a profound TOC, and are easy to process by standard lithography like most polymers, but furthermore feature a considerably high thermal and chemical stability which even equates to inorganic materials such as glasses [15,16].

In this report, we therefore demonstrate the fabrication of embedded channel waveguide Bragg gratings written directly into thin layers of these Ormocer^®^ hybrid polymers. In the employed approach, both waveguide as well as the Bragg grating are written simultaneously by applying a single writing step technique that is based on the static phase mask technology and KrF excimer laser irradiation, and has already been proven to be a highly efficient concept for the fabrication of waveguide Bragg gratings in bulk polymers, as demonstrated by the authors and others elsewhere [17]. Since Ormocer^®^s feature considerably high TOCs as well as a high thermal stability, they represent a promising material for the fabrication of temperature sensors. Therefore, we present and discuss the response behavior and sensitivity of the accordingly fabricated devices on temperature changes. However, some polymer materials exhibit distinct cross-sensitivities to the temperature accompanying relative humidity [18,19]. Consequently, the influence of a changing relative humidity on the fabricated Bragg gratings is furthermore investigated and discussed.

## 2. Sensor Fabrication

**Figure 1:**
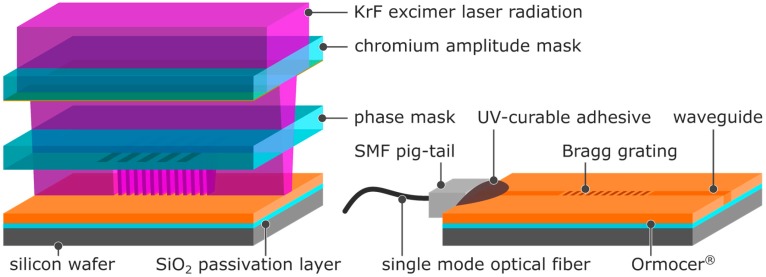
Schematic illustration of the waveguide Bragg grating fabrication process (note: the illustration shows an exploded-view drawing. The actual fabrication takes place with the chromium amplitude mask and the phase mask put together and being in direct contact with the surface of the Ormocer^®^ layer).

The multilayer substrate of the fabricated sensor (Figure 1) consists of a p/Boron silicon wafer of 76.2 mm width that contains a thermally grown 2 µm-thick SiO${}_{2}$ passivation layer which serves as an underclad to the waveguide. 

For an improved adhesion of the Ormocer^®^s, a thin layer of the adhesion promoter OrmoPrime^®^08 (micro resist technology, applied as purchased) was spin-coated on to the wafer at 4000 rpm and cured on a hotplate set to 150 ${}^{\circ }$C for 5 min. As waveguide material, the inorganic–organic Ormocer^®^ hybrid polymers OrmoComp^®^, OrmoCore, and OrmoClad were applied (micro resist technology, applied as purchased). These hybrid polymers are specially designed for the fabrication of waveguides and micro-optics. While OrmoCore (*n*${}_{D}$ = 1.555) and OrmoClad (*n*${}_{D}$ = 1.537) are generally designed for UV-lithography of waveguides and cladding, respectively, OrmoComp^®^ (*n*${}_{D}$ = 1.520) is designed for UV-molding of coupler and micro-optics. All three Ormocer^®^s feature a high optical transparency in the VIS and IR spectral range and ensure a thermal stability up to 270 ${}^{\circ }$C [20,21]. The Ormocer^®^s were spin-coated onto the silicon wafers at 6000 rpm for OrmoComp^®^ and OrmoCore and at 5500 rpm for OrmoClad and cured by flood UV-exposure at 1200 mJ·cm${}^{-2}$, followed by a 10 min post-exposure bake at 150 ${}^{\circ }$C. In order to remove any uncured material (as it occurs in the form of an inhibition layer if the UV-exposure is performed in the presence of oxygen), the substrates were subsequently developed in 1-methoxy-2-propanol acetate for 10 s, rinsed with 2-propanol, and then gently blow-dried using nitrogen. The preparation of the substrate was completed by a 3-h hardbake at 150 ${}^{\circ }$C. By this measure, Ormocer^®^ films with a thickness of 6 ± 2 µm as determined by m-line spectroscopy were achieved. For better handling, the substrates were cleaved into single chips of 15 mm length and 10 mm width by laser dicing. As illustrated in Figure 1, the waveguide and Bragg grating were inscribed into the Ormocer^®^ layers simultaneously using a single writing step technique. In this approach, a waveguide defining chromium amplitude mask which features a waveguide aperture of 27 µm width was put on top of a Bragg grating defining phase mask that in turn was put in direct contact with the surface of the respective Ormocer^®^ layer. The applied phase masks feature a grating period of $\Lambda_{d}$ = 1020 nm for the fabrication of Bragg gratings in OrmoComp^®^ and $\Lambda_{d}$ = 1008 nm for the fabrication of Bragg gratings in OrmoCore and OrmoClad. As a light source, a 248 nm KrF excimer laser was used with the laser beam being shaped into a rectangular geometry using cylindrical lenses and guided through the stacked masks onto the substrate. Thus, while the direct contact of the phase mask to the Ormocer^®^s provides a grating period of Λ = $\Lambda_{d}$/2, the considerable distance of the amplitude mask to the substrate (as a consequence of the intervening phase mask) leads only to a projection of the amplitude mask’s aperture and resulted in a waveguide width of approx. 15 µm. By applying an excimer laser fluence of 3 J·cm${}^{-2}$, 1 J·cm${}^{-2}$, and 5 J·cm${}^{-2}$ (at a single pulse fluence of 10 mJ·cm${}^{-2}$ and a repetition rate of 20 Hz) for OrmoComp^®^, OrmoCore, and OrmoClad, respectively, both waveguide and Bragg grating were inscribed into the hybrid polymers simultaneously. Subsequently to the inscription process, a single-mode optical fiber pig-tail comprising a standard FC/APC connector was butt-coupled and bonded to the waveguide facet using a UV-curable adhesive.

## 3. Experimental

**Figure 2:**
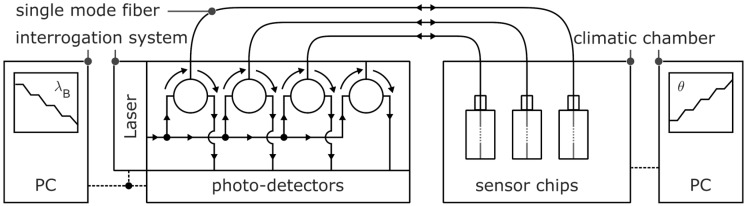
Schematic illustration of the experimental setup.

For the detection and tracking of the sensor chips’ reflected Bragg wavelengths, the waveguide Bragg gratings were connected to a four-channel source and detector interrogation system (sm125, micron optics) which operates at telecom wavelengths between 1510 nm and 1590 nm with a resolution of 1 pm and allows simultaneous tracking of multiple Bragg reflections at a sampling rate of 2 Hz. For the determination of the temperature and humidity sensitivity, a climatic chamber (WKL 64-40, Weiss Technik) was applied in which the fabricated and connected sensor chips were placed (Figure 2). The temperature and humidity sensitivity of the Bragg gratings were then investigated by detecting and tracking the respective reflected Bragg wavelengths of the sensors while both the temperature and the relative humidity were varied accordingly.

In order to gain a more profound understanding of the impact a possible water absorption (e.g., due to relative humidity) has on the material’s refractive index and volume, both of which would affect the reflected Bragg wavelength $\lambda_{B}$ (c.f. condition (1)), m-line measurements (2010/M, Metricon) of appropriately treated samples were performed. In this approach, a substrate of each Ormocer^®^ (as multilayer configuration without waveguide and Bragg grating) was stored in deionized water for more than 12 h, and after its retrieval immediately dabbed and mounted into an m-line spectroscope and repetitively measured for approximate 90 min. After this treatment, possibly absorbed water should slowly desorb and the measurement results, although contrary, should indicate the effect of an accordingly occurring water absorption.

Additionally, numerical beam-propagation simulations of appropriately designed asymmetric embedded channel waveguides were performed (RSoft™ BeamPROP™, Synopsys^®^) to classify the experimentally investigated Bragg reflections with regard to the modes and polarizations assigned to them. 

## 4. Results and Discussion

**Figure 3:**
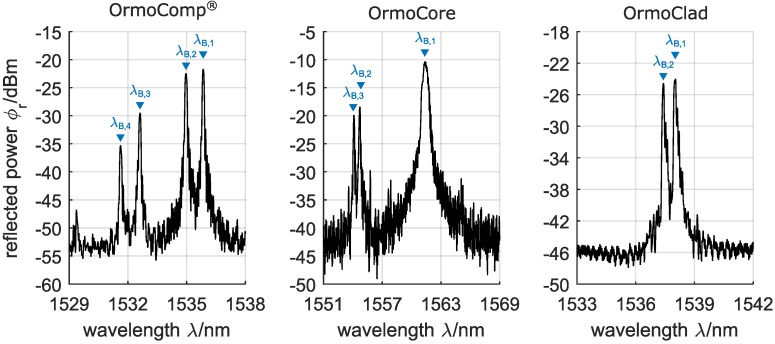
Reflection spectra of embedded channel waveguide Bragg gratings in OrmoComp^®^, OrmoCore, and OrmoClad, fabricated with a single writing step technique.

Embedded channel waveguide Bragg gratings were fabricated in the Ormocer^®^ hybrid polymers OrmoComp^®^, OrmoCore, and OrmoClad in a single writing step technique. The reflection spectra of accordingly fabricated devices are depicted in Figure 3.

The reflection spectra of the Bragg gratings exhibit multiple reflection peaks $\lambda_{B,i}$, which can be dedicated firstly to a multi-mode distribution within the waveguides and secondly to a geometrical as well as stress-induced birefringence, where the latter causes a splitting of the Bragg reflection peaks. Numerical simulations of the propagating modes’ effective refractive indices indicate that the Bragg reflections at higher wavelengths can be assigned to the propagating fundamental mode, while Bragg reflections at lower wavelengths can be dedicated to higher order modes (a more detailed description of the simulation-based Bragg peak assignment can be found in the appendix). However, the distribution and allocation of higher order modes is strongly depending on the exact waveguide dimensions and the coupling-position, which is only conditionally controllable due to the manually performed coupling and bonding process. With regard to the birefringence (which causes a splitting of the Bragg reflections), the performed simulations revealed that the reflection peak at the lower wavelength position can be assigned to the propagating mode’s TM-polarization, while the reflection peak at the higher wavelength position can be assigned to the propagating mode’s TE-polarization. Consequently, the reflection peaks $\lambda_{B,1}$ and $\lambda_{B,2}$ of the waveguide Bragg grating in OrmoComp^®^ can be assigned to the fundamental mode’s TE-polarization and TM-polarization, respectively, while the reflection peaks $\lambda_{B,3}$ and $\lambda_{B,4}$ can be assigned to the higher order mode’s TE-polarization and TM-polarization, respectively. In case of the Bragg grating in OrmoCore, $\lambda_{B,1}$ is the result of a superposition of the fundamental mode’s TE- and TM-polarization, while $\lambda_{B,2}$ and $\lambda_{B,3}$ can be assigned to the higher order mode’s TE-polarization and TM-polarization, respectively. For the Bragg grating in OrmoClad, single mode distribution can be found, where $\lambda_{B,1}$ and $\lambda_{B,2}$ can be assigned to the fundamental mode’s TE-polarization and TM-polarization.

**Figure 4:**
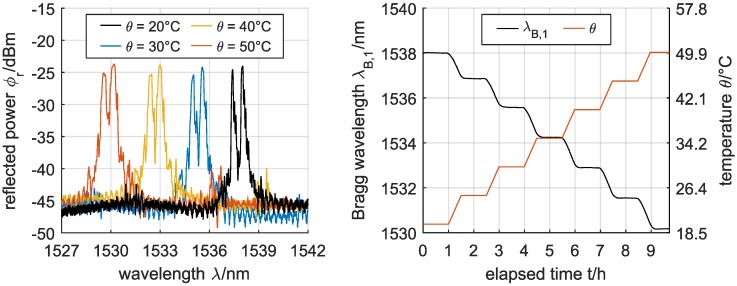
Reflection spectra of a waveguide Bragg grating in OrmoClad (**left**) and the correspondingly detected and tracked Bragg wavelength shift of $\lambda_{B,1}$ depending on the temperature at a relative humidity of 30 % (**right**).

All fabricated Bragg gratings exhibited narrow-band reflection peaks with a −3 dB-bandwidth of down to Δλ = 95 pm as well as sufficient peak heights of $\Delta \phi_{r}$ ≥ 20 dB, and are thus well suited for sensor applications that require a reliable detection and tracking of the reflection peaks. As an example, the spectral response of an embedded channel waveguide Bragg grating fabricated in OrmoClad to an increasing temperature θ is shown in Figure 4.

**Figure 5:**
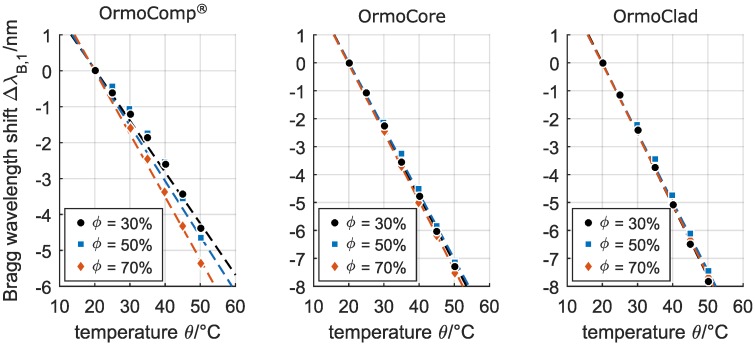
Linear dependence of the buried channel waveguide Bragg gratings in OrmoComp^®^, OrmoCore, and OrmoClad on the temperature for a relative humidity ϕ of 30 %, 50 %, and 70 %.

The reflected Bragg wavelengths of all fabricated devices show a quasi-instantaneous response to temperature changes, where, due to the negative thermo-optic coefficient of the hybrid polymers, a temperature increase of the waveguide Bragg gratings results in a decrease of the material’s refractive index and thus in a distinct blue-shift of the reflected Bragg wavelengths. By depicting the respective wavelength shifts of the Bragg gratings as a function of the applied temperature θ and relative humidity ϕ (Figure 5), a linear relationship of the fabricated devices towards the temperature can be found. Even though Figure 5 only shows the wavelength shifts of the $\lambda_{B,1}$ reflection peaks of the embedded channel waveguide Bragg gratings, all peaks of one Bragg grating (regardless of the propagating mode and its polarizations) exhibit the same relative shifts and thus feature the same sensitivity.

While the sensitivity of the Bragg grating fabricated in OrmoComp^®^ shows a noticeable increase with increasing relative humidity with −145 pm/${}^{\circ }$C (*R*${}^{2}$ = 0.98), −155 pm/${}^{\circ }$C ($R^{2}$ = 0.95), and −175 pm/${}^{\circ }$C ($R^{2}$ = 0.97) for 30 %, 50 %, and 70 %, respectively, the sensitivities of the Bragg gratings fabricated in OrmoCore and OrmoClad are consistent for all applied relative humidity levels with approximate −240 ± 5 pm/${}^{\circ }$C ($R^{2}$ ≥ 0.99) and −252 ± 3 pm/${}^{\circ }$C ($R^{2}$ ≥ 0.99), respectively. Consequently, the fabricated buried channel waveguide Bragg gratings in Ormocer^®^ hybrid polymers feature temperature sensitivities of up to 20-times the sensitivity of silica-based Bragg gratings. Taking into account the spectral resolution of the applied interrogation system of 1 pm and a signal noise of 2 pm, the waveguide Bragg gratings in OrmoCore and OrmoClad feature reliable detection limits of 2.5 × 10${}^{-2}$ ${}^{\circ }$C and 2.4 × 10${}^{-2}$ ${}^{\circ }$C, respectively.

**Figure 6:**
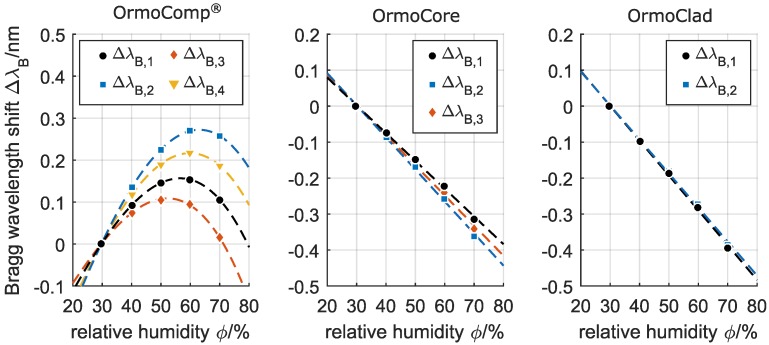
Dependence of the planar waveguide Bragg gratings in OrmoComp^®^, OrmoCore, and OrmoClad on the relative humidity at a temperature θ of 30 ${}^{\circ }$C.

The dependency of the devices’ reflected Bragg wavelengths on a changing relative humidity in the range of 30 % to 70 % at standard atmosphere and a constant temperature of 30 ${}^{\circ }$C (which corresponds to a dependency on the absolute humidity in the range of 9 × 10${}^{-3}$ g/L to 21.5 × 10${}^{-3}$ g/L) is depicted in Figure 6.

It is striking that the reflected Bragg wavelengths of the embedded channel waveguide Bragg grating in OrmoComp^®^ show a non-linear red-shift at an increasing relative humidity, while the reflected Bragg wavelengths of the Bragg gratings fabricated in OrmoCore and OrmoClad exhibit a linear blue-shift. M-line spectroscopic analyses of the hybrid polymer substrates indicate a slight refractive index decrease with an increasing water absorption for all processed Ormocer^®^s. However, exclusively for the OrmoComp^®^, a swelling of the whole hybrid polymer layer with increasing water absorption is distinguishable. Since water has a lesser refractive index than the applied Ormocer^®^s, an absorption of water results in a reduction of the effective refractive index of the guided mode and thus in a decrease of the reflected Bragg wavelength, as observable for the Bragg gratings fabricated in OrmoCore and OrmoClad. On the other hand, a swelling of the Ormocer^®^ layer leads to an increasing waveguide height and an elongation of the Bragg grating period, and thus to an increase of the reflected Bragg wavelength. This effect, which is only present for the Bragg grating in OrmoComp^®^, counteracts the simultaneously occurring refractive index reduction and leads to the observable non-linear shift of the respective Bragg wavelengths on an increasing relative humidity.

Furthermore, a variation of the sensitivities for the different reflected Bragg wavelengths can be observed. Here, the higher order mode-caused reflection peaks show a slightly enhanced blue-shift compared to the fundamental mode-caused reflection peaks, whereas the wavelength shifts are generally more pronounced for the respective TE-polarizations. The wavelength shifts of the Bragg grating in OrmoCore were found to be −7.7 pm/%, −8.9 pm/%, and −8.3 pm/% (all at an $R^{2}$ ≥ 0.99) for $\lambda_{B,1}$, $\lambda_{B,2}$, and $\lambda_{B,3}$, respectively, and the wavelength shifts of the Bragg grating in OrmoClad were found to be −9.7 pm/% and −9.5 pm/% (both at an $R^{2}$ ≥ 0.99) for $\lambda_{B,1}$ and $\lambda_{B,2}$, respectively. However, compared to the high temperature sensitivities, the dependence of the reflected Bragg wavelengths on the relative humidity is considered as rather feeble.

## 5. Conclusions

The fabrication of embedded channel waveguide Bragg gratings in the Ormocer^®^ hybrid polymers OrmoComp^®^, OrmoCore, and OrmoClad by an efficient and easy to apply single writing step technique based on phase mask technology and excimer laser irradiation was demonstrated. All fabricated Bragg gratings exhibited well-defined Bragg reflections within the telecom wavelengths range which are (rated by their bandwidth and peak height) well suited for sensing applications that require a reliable detection and tracking of the respective Bragg wavelengths. We furthermore investigated the sensitivities of the thus-fabricated waveguide Bragg gratings towards temperature and moreover to the accompanying relative humidity. Here, all three materials provide the foundation for highly sensitive Bragg grating temperature sensors that facilitate sensitivities of −160 ± 15 pm/${}^{\circ }$C, −240 ± 5 pm/${}^{\circ }$C, and −252 ± 3 pm/${}^{\circ }$C for OrmoComp^®^, OrmoCore, and OrmoClad, respectively, which represents a 12-to-20-fold sensitivity increase compared to bare silica-based FBGs. Alternative polymer-based Bragg grating concepts for temperature sensing — such as polymer-coated FBGs [9,10], Bragg gratings fabricated in polymer optical fibers [11,12], or waveguide Bragg gratings written in polymers [13,14] — yield sensitivities of three-to-ten-times the sensitivity of bare silica-based FBGs. For the Bragg gratings fabricated in OrmoComp^®^, a humidity-dependent temperature sensitivity was found which increased with increasing relative humidity. However, the Bragg gratings fabricated in OrmoCore and OrmoClad provided a consistent temperature sensitivity which was unaffected by the relative humidity. Therefore, and with respect to a more reliable assignability of an observed wavelength shift to the respective underlying temperature change, the hybrid polymers OrmoCore and OrmoClad are favorable for the fabrication of temperature-sensitive Bragg gratings. The dependencies of the hybrid polymer based embedded waveguide Bragg gratings on the relative humidity were found to be −8.3 ± 0.6 pm/% for the Bragg grating in OrmoCore and −9.6 ± 0.1 pm/% for the Bragg grating in OrmoClad, and are considered as rather feeble compared to the respective high temperature sensitivities. The reflected wavelengths of the Bragg grating in OrmoComp^®^ on the other hand exhibited non-linear red-shifts which can be attributed to a water absorption-caused refractive index decrease and simultaneous swelling of the material. These mechanisms have a contrary effect on the reflected Bragg wavelength, and thus result in an observable non-linearity that makes the prediction and accounting of any relative humidity-caused cross-sensitivities a difficult task. In this respect, the noticeable dependence of the OrmoComp^®^-based Bragg gratings on the relative humidity clearly shows that an application of this hybrid polymer is not suitable for the fabrication of Bragg grating-based temperature sensors. On the other hand, the Ormocer^®^s OrmoCore and OrmoClad represent a promising polymer material for the fabrication of Bragg grating-based temperature sensors, since the accordingly fabricated devices exhibit a very high temperature sensitivity, a minor but linear dependence on the relative humidity, and feature a high thermal as well as chemical stability.

## Appendix A. Details on the Simulation-Based Bragg Peak Assignment

Beam propagation simulations of an appropriately designed asymmetric buried channel waveguide structure are performed using Synopsys^®^ RSoft™ BeamPROP™ and are furthermore experimentally cross-checked.

In order to assign the experimentally determined Bragg wavelengths, the effective indices of the propagation modes and their respective polarizations are simulated. The results show, that for all Ormocer^®^s (which primarily differ by their refractive indices), the quasi-TE polarization of the fundamental mode exhibits the highest effective refractive index followed by the quasi-TM polarization of the fundamental mode. The simulations furthermore show, that higher order modes generally exhibit lesser effective refractive indices. However, the exact order of the higher order modes TE- and TM-polarizations’ effective indices is strongly depending on the exact waveguide dimensions and the coupling-position. Thus, based on only these simulations, a reliable conclusion on which higher order mode and which respective polarization causes a certain reflection at lower wavelength cannot be made. In order to cross-check the simulated results and to clarify the polarization affiliation of the higher order modes, the fabricated waveguide Bragg gratings are connected to an interrogation system without polarization control. By bending the fiber pig-tail connected to the respective Bragg gratings, stress-induced polarization of the guided light is aroused, which is noticeable by an attenuation of either the TE-polarization caused reflection peaks or the TM-polarized reflection peaks for different bending directions. Here, for all three fabricated waveguide Bragg gratings, either all right-located split-up Bragg peaks or all left-located split-up Bragg peaks are attenuated simultaneously. Thus, the distribution of the TE- and TM-polarization caused reflection peaks - with respect to their location in terms of left- or right-located - is the same for all experimentally determined split-up Bragg peaks.

Further simulations and experiments are performed in order to confirm the simulation results of the fundamental mode’s TE-polarization to exhibit the highest effective refractive index. In these additional investigations, the refractive index of the surrounding material is altered. In the experimental investigations, both, the TE- and TM-polarization caused Bragg reflection peaks are stimulated in equal measure and water is applied to the surface of the sensor (which changes the surroundings refractive index from *n* = 1 to *n* = 1.33). While the simulation show a more profound effective refractive index increase of the TM-polarized reflection at an increasing surrounding’s refractive index than the TE-polarized reflection, the experimental investigations show a more profound Bragg wavelength shift of the left-located split-up Bragg peak at an increasing surrounding’s refractive index than the right-located split-up Bragg peak. Therefore, for the fundamental mode as well as the higher order modes it is confirmed, that all left-located split-up peaks can be attributed to the TM-polarization and all right-located split-up peaks can be attributed to the TE-polarization.
